# The Effects of Partner Extraversion and Agreeableness on Trust

**DOI:** 10.1177/01461672221086768

**Published:** 2022-04-28

**Authors:** Olga Stavrova, Anthony M. Evans, Ilja van Beest

**Affiliations:** 1 Tilburg University, The Netherlands

**Keywords:** trust, extraversion, agreeableness, trustworthiness, group work, impression management

## Abstract

Existing research has documented the social benefits (i.e., higher popularity and liking) of extraversion and agreeableness. Do these positive reputational consequences extend to social dilemma situations that require trust? We found that people do not trust extraverts more than introverts. Instead, people’s trust decisions are guided by their partner’s level of agreeableness. In a trust game (Studies 1 and 2), individuals were more likely to trust a partner who was described as agreeable (vs. disagreeable); and, in a laboratory study of work groups, participants trusted more (vs. less) agreeable group members (Study 3). Individuals anticipated others’ preferences for agreeable partners and tried to come across as more agreeable, but not more extraverted, in social dilemmas (Study 4). These findings suggest that the social benefits of agreeableness (but not extraversion) extend to social interactions involving trust and highlight the importance of target personality traits in shaping trust decisions.

When interacting with strangers for the first time, people quickly and effortlessly assess their personality traits (Borkenau et al., 2004). How do these inferences affect individuals’ behavior toward others, such as the decisions to trust and cooperate? Existing research on the reputational consequences of personality has highlighted the social benefits of extraversion: Compared with introverts, extraverts are more popular, likable, and are ascribed a higher status in groups (Feiler & Kleinbaum, 2015; Judge et al., 2002). Does the popularity of extraverts extend to the social dilemma of trust?

In this research, we examined how partner extraversion influences trust at zero-acquaintance. We compared the effect of partner extraversion with the effect of partner agreeableness—the dimension of the Big Five that is most central to trustworthiness and prosociality more generally. We examined how perceptions of extraversion and agreeableness (manipulated experimentally or naturally observed in interaction partners) influence expectations of trust behavior. Are people more willing to trust in extraverted (vs. introverted) and agreeable (vs. disagreeable) partners? And do people anticipate preferences for agreeable and extraverted partners and try to come across as more agreeable and extraverted in social dilemma situations? This research adds to our understanding of how lay beliefs of personality influence the development of trust and cooperation among strangers.

## The Role of Personality in Person Perception

Personality traits play an important role in person perception (Back et al., 2011). When it comes to social interactions, two traits typically stand out: extraversion and agreeableness (McCrae & Costa, 1989; Tov et al., 2016). Extraversion describes individuals in terms of their tendency to be the center of attention in social interactions, whereas agreeableness classifies individuals as more (vs. less) helpful and responsive to others’ needs. In social perception literature, these dimensions of individual differences are described in terms of agency (also referred to as dominance or assertiveness) and warmth (also referred to as affiliation or communion; Imhoff & Koch, 2017; McCrae & Costa, 1989; Trapnell & Wiggins, 1990). Warmth has been recently shown to encompass two further distinct dimensions: sociability (friendliness, likability) and morality (honesty, trustworthiness; Brambilla et al., 2011). Whereas agreeableness is conceptually related to sociability and morality, extraversion reflects sociability and agency (Abele et al., 2016; Depue & Collins, 1999; DeYoung et al., 2007; Do & Minbashian, 2014). Agency and warmth (including sociability and morality) guide social perception and impression formation (Abele et al., 2008; Fiske, 2018). Multiple studies have confirmed that individuals’ overall judgments of others are based on their evaluations of others’ assertiveness and dominance, friendliness and kindness, and, in particular, moral character (Abele et al., 2008; Brambilla et al., 2019, 2011; Brambilla & Leach, 2014). Therefore, in exploring the effect of partner personality on trust, we focused on individual differences in extraversion and agreeableness.

## Extraversion

Previous research has highlighted the role of extraversion in determining who is liked and respected by peers. Among adolescents, extraverts tend to have higher peer acceptance and sociometric popularity (Jensen-Campbell et al., 2002; van der Linden et al., 2010; Wolters et al., 2014). The sociometric advantage of extraverts persists throughout adulthood: Extraversion is associated with larger networks of friends (Asendorpf & Wilpers, 1998; Lönnqvist & große Deters, 2016; Shen et al., 2015) and a higher ascribed status in face-to-face groups (Anderson et al., 2001; Anderson & Kilduff, 2009). Based on these findings, extraverts may be more likely to be trusted and preferred as cooperation partners than introverts. Yet other research casts doubt on this prediction.

Although extraverts have larger social networks, they tend to maintain relatively superficial relationships with a lot of people, often failing to develop deeper ties and the emotional closeness that build the foundation of trust (Judge et al., 2009; Pollet et al., 2011). In addition, the assertiveness and dominance orientation of extraverts might undermine their desirability as interaction partners in highly interdependent settings that require trust and cooperation, such as social dilemma situations. Indeed, observations of assertiveness and excitement seeking in others are associated with attributions of higher competitiveness (rather than cooperation; Cooper et al., 2015). Similarly, extraversion has been associated with more competitive behaviors in auction games (Fong et al., 2021). Further studies linked extraversion to conflict-proneness and aggression. For example, living with a more (vs. less) extraverted roommate has been associated with a higher level of interpersonal conflict (Bono et al., 2002) and a recent meta-analysis revealed a positive association between extraversion and bullying (Mitsopoulou & Giovazolias, 2015). In work settings, although a higher level of enthusiasm and confidence allow extraverts to attain leadership positions initially, over time, extraversion is associated with status losses, as extraverts’ actual contributions to group tasks do not match group members’ initial expectation (Bendersky & Shah, 2013)—a finding also observed for other traits related to extraversion, such as narcissism (Back et al., 2013).

Finally, the literature on personality and trustworthy behaviors (e.g., in economic games) suggests that extraverts are not necessarily more likely to behave in a trustworthy manner than introverts (Müller & Schwieren, 2020; Thielmann & Hilbig, 2015; Zhao & Smillie, 2015). Hence, to the extent that people are able to connect extraversion and observations of trustworthy behaviors in others, they should not expect more trustworthy behaviors from extraverts. Indeed, studies on the drivers of positive social impressions (such as liking and respect) showed that perceptions of morality (e.g., honesty, compassion, and fairness) are more central to liking than perceptions of more agentic traits (e.g., assertive, influential; Goodwin et al., 2014; Hartley et al., 2016) and, whereas moral traits are always evaluated positively, traits associated with sociability (e.g., friendliness) can, depending on the target morality, lead to positive *or* negative evaluations (Landy et al., 2016).

Taken together, these different lines of literature suggest that extraversion might have either positive or negative interpersonal effects in social dilemma situations.

## Agreeableness

Agreeableness can be expected to represent a highly desired trait for interaction partners in social dilemmas: after all, agreeable individuals are described as warm, helpful, and cooperative (Goldberg, 1992). Agreeableness is associated with real-life prosocial behaviors, such as helping and volunteering (Carlo et al., 2005; Graziano et al., 2007), as well as prosocial behaviors at work (organizational citizenship behavior; Bourdage et al., 2012). In teams, more (vs. less) agreeable members are more likely to take on “social roles,” including conflict mediation, building group solidarity and cohesion, and satisfying the emotional needs of others (Stewart et al., 2005).

Not surprisingly, agreeableness promotes likability (van der Linden et al., 2010). For example, higher levels of agreeableness predict being liked by peers, a lower likelihood of victimization, and higher chances of being selected as friends (Jensen-Campbell et al., 2002; Selfhout et al., 2010; Stopfer et al., 2013, 2014). In the work context, people perceive more (vs. less) agreeable members of their work team as more trustworthy (Naber et al., 2018). Also, revealing partner agreeableness scores boosts cooperation in more (vs. less) agreeable dyads (Drouvelis & Georgantzis, 2019). Hence, we expected individuals to show more trust in more (vs. less) agreeable others.

## The Present Research

We examined the partner-level effects of extraversion and agreeableness on individuals’ trust decisions. Specifically, we tested whether people see others’ agreeableness and extraversion as cues to their trustworthiness and are more likely to trust more (vs. less) agreeable and more (vs. less) extraverted others. We also explored whether people are aware of the importance of agreeableness and extraversion in trustworthiness judgment, and whether they try to come across as more agreeable and extraverted than they actually are.

These questions were examined using (partially) incentivized economic games, online experiments, and laboratory observation of work groups. Studies 1, 2, and 4 were preregistered; Study 3 was exploratory. All studies’ materials, data, and computer code can be downloaded from https://osf.io/b68cs/.

## Study 1

Study 1 examined how partner agreeableness and extraversion influence trust at zero acquaintance. Participants played a trust game with a partner described as either agreeable versus disagreeable, and extraverted versus introverted. We hypothesized that higher levels of agreeableness will be associated with a higher likelihood to be trusted by others. Our predictions regarding the effect of extraversion were guided by the literature on positive reputational consequences of extraversion (e.g., Lönnqvist & große Deters, 2016; Shen et al., 2015), resulting in the expectation of higher trust toward more (vs. less) extraverted targets.

Measures, data collection, and analyses were preregistered (https://aspredicted.org/at8bt.pdf). We did not deviate from the preregistered analysis plan.

### Method

#### Design and procedure

The study used a 2 (*Trustee Agreeableness*: high vs. low) × 2 (*Trustee Extraversion*: high vs. low) between-subjects design. Participants learned that they would make decisions that would affect their own payoffs and the payoffs of another participant. The payoff for each decision was given in GBP and, at the end of the study, we paid out 10 randomly selected participants. As we were interested in trustors’ decisions only, all participants were assigned to the role of trustor. To ensure a fair treatment (despite deception), participants who were selected for payoff received the amount that they would have received if their partner was trustworthy (i.e., sent back 50% of the total amount, which represents the most common trustee decision).

Participants read that they would be matched with another participant of this study in a decision-making task (the trust game): Participants played the role of Person 1 (the trustor) and the other participant played the role of Person 2 (the trustee). Person 1 started with £1 and could choose to transfer it to Person 2 (or to keep the money). If Person 1 transferred £1, then it was tripled and given to Person 2 (so Person 2 had a total of £3). Person 2 could then choose how much they wanted to transfer back to Person 1 (between £0 and £3).

To check participants’ understanding of the game, we asked them what would happen if they transferred £1 and Person 2 transferred half of the total amount back to them (three response options: “You earn £1.5, Person 2 earns £1.5”; “You earn £0, Person 2 earns £3”; and “You earn £0, Person 2 earns £1”).

Afterward, participants were provided with additional information regarding their interaction partner. They read that Person 2’s name was Cory, and that Cory was either agreeable (or disagreeable) or extraverted (or introverted). Specifically, we constructed four profiles reflecting the different combinations of agreeableness and extraversion: “disagreeable and extraverted,” “disagreeable and introverted,” “agreeable and extraverted,” and “agreeable and introverted.” That way, we could examine the effect of one trait (e.g., agreeableness) independent of the effect of the other (e.g., extraversion). To construct the profiles, we used items from the International Personality Item Pool (IPIP) scales for agreeableness and extraversion (Goldberg, 1992; see Table 1 for the exact wording). We also varied whether the extraversion- versus agreeableness-related information was presented first. Trait order had no significant effect on the trust decision and did not significantly interact with other factors (all *p*s > .15), so for brevity reasons, we will present the analyses without this factor.

**Table 1.** Manipulation of Trustee Personality, Study 1.

	High extraversion	Low extraversion
High agreeableness	Cory doesn’t mind being the center of attention and talks to a lot of different people at parties. Cory makes people feel at ease, sympathizes with their feelings, and has a soft heart.	Cory doesn’t talk a lot, is quiet around strangers, and usually keeps in the background. Cory makes people feel at ease, sympathizes with their feelings, and has a soft heart.
Low agreeableness	Cory doesn’t mind being the center of attention and talks to a lot of different people at parties. Cory is generally not interested in other people’s problems and feels little concern for them.	Cory doesn’t talk a lot, is quiet around strangers, and usually keeps in the background. Cory is generally not interested in other people’s problems and feels little concern for them.

Participants were then asked to indicate whether they would transfer their endowment (£1) to Cory or not (1 = *transfer*, 0 = *no transfer*). This constituted a measure of behavioral trust and represented our dependent variable.

Finally, using a 5-point scale, participants completed a manipulation check by rating Cory on the dimensions of agreeableness (“Cory feels little concern for other people’s problems” [reverse-coded], “Cory sympathizes with other people’s feelings,” averaged into a measure of perceived agreeableness, *r* = .61, *p* < .001) and extraversion (“Cory keeps in the background” [reverse-coded], “Cory doesn’t mind being the center of attention,” averaged into a measure of perceived extraversion, *r* = .59, *p* < .001). For (preregistered) exploratory analysis,^1^ we also asked participants to indicate to what extent they thought that Cory had “a coherent personality.” At the end, participants filled in basic sociodemographic information.

#### Participants

We used a power analysis with G*Power 3.1 (Faul et al., 2009) to determine the sample size. To be able to detect medium effects (we focused on main effects, as we did not have any predictions regarding the interaction effect; odds ratio [OR] = 2.46, logistic regression; α = .05, two-tailed test, 80% power^2^), we aimed at collecting at least 200 participants. To compensate for participants failing the question testing the comprehension of the rules of the trust game, we decided to recruit an additional 100 participants. Hence, a total of 300 participants completed the study on Prolific Academic. Of them, 258 responded correctly to the comprehension question and constituted our final sample (*M*_age_ = 30.12, *SD*_age_= 10.05; 53.1% male).

### Results

#### Manipulation check

A 2 × 2 multivariate analysis of variance (MANOVA; with perceptions of agreeableness and extraversion as dependent variables) showed that agreeable trustees were perceived as being more agreeable (*M* = 3.97, *SD* = 0.83) than disagreeable trustees (*M* = 1.87, *SD* = 0.88), *F*(1, 252) = 388.56, *p* < .001, $\eta_{p}^{2}$ = .61, 95% confidence interval (CI) = [.53, .66], and that extraverted trustees were perceived as more extraverted (*M* = 4.14, *SD* = 0.98) than introverted trustees (*M* = 1.93, *SD* = 0.84), *F*(1, 252) = 372.01, *p* < .001, $\eta_{p}^{2}$ = .60, 95% CI = [.52, .65]. Trustee agreeableness did not significantly affect participants’ perception of trustee extraversion, *F*(1, 252) = .13, *p* = .72, and trustee extraversion did not significantly affect participants’ perception of trustee agreeableness, *F*(1, 252) = 2.04, *p* = .15. The interaction between trustee agreeableness and trustee extraversion was not significant either, *F*(2, 251) = 0.27, *p* = .76. In addition, perceived extraversion was unrelated to perceived agreeableness (*r* = −.01, *p* = .85). In short, our manipulations of trustee agreeableness and extraversion were both independently successful.

#### Trust decisions

To examine whether trustee extraversion and agreeableness affected participants’ trust decisions, we used logistic regression with participants’ trust decision (1 = *trust*, 0 = *no trust*) as dependent variable. In Step 1, we entered the two manipulated factors—trustee extraversion and agreeableness—as predictors (*high* = 1, *low* = 0).

**Figure 1.**
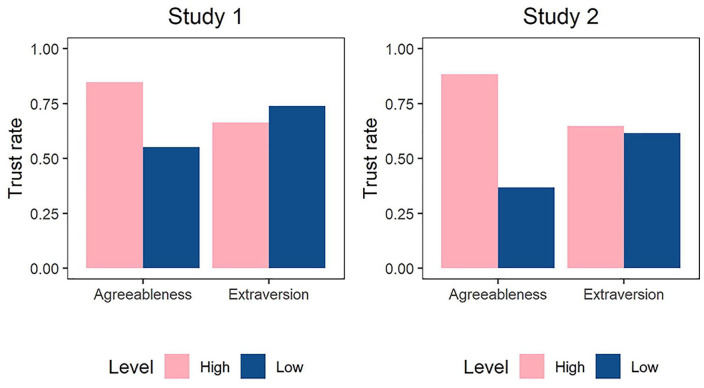
Trust rate depending on target agreeableness and extraversion, Study 1 (left) and Study 2 (right). *Note*. Trust rate reflects the percentage of the participants in each condition who decided to trust the target.

Participants were more likely to trust agreeable than disagreeable trustees (OR = 4.68, *p* < .001, 95% CI = [2.58, 8.50]). That is, an agreeable trustee’s chance of being trusted was more than 4 times higher compared with a disagreeable trustee’s chance. In contrast, trustee extraversion was not significantly related to participants’ trust (OR = .62, *p* = .106, 95% CI = [0.35, 1.11]); if anything, participants were slightly more likely to trust introverted (vs. extraverted) trustees. In Step 2, we added an interaction between trustee agreeableness and extraversion. The interaction was not significant (OR = 1.74, *p* = .36, 95% CI = [0.53, 5.72]), suggesting that agreeable trustees were more likely to be trusted than disagreeable trustees regardless of their level of extraversion. The share of participants who trusted the target in each condition is shown in Figure 1.

### Discussion

Study 1 showed that participants’ trust decisions were driven by their interaction partner’s level of agreeableness, rather than extraversion. Hence, despite extraverts’ general popularity in social settings demonstrated in the literature so far (e.g., Anderson & Kilduff, 2009), extraversion does not seem to be an asset in the social dilemma of trust.

## Study 2

Study 2 was designed to replicate and extend the results of Study 1 in two ways. First, as noticed by an anonymous reviewer, the description of the extraverted target in Study 1 arguably did not fully reflect the characteristics that drive the likability of extraverts (e.g., being outgoing and sociable). To address this issue, we developed a new manipulation of target agreeableness and extraversion. In a pretest, we asked participants to rate 10 agreeableness and 10 extraversion items (Big Five Inventory [BFI]-44; John & Srivastava, 1999) on likability; we then selected the three items from each scale that achieved similar likability scores and used them to create a description of agreeable and extraverted targets in the main study. Second, given that trust and liking are closely related, we sought to test, in Study 2, whether the effect of target agreeableness on being trusted holds above and beyond just liking. To achieve this, we asked participants to rate the target on both trustworthiness and liking, and tested whether perceived trustworthiness accounts for the effect of target agreeableness on trust above and beyond liking.

Measures, data collection, and analyses were preregistered (https://aspredicted.org/te5zi.pdf). We did not deviate from the preregistered analysis plan.

### Method

#### Pretest

The pretest was not included in the preregistration. We recruited 101 individuals on Prolific. Fourteen did not pass an attention check question (“To monitor data quality, select the middle of the scale here”) and were removed, resulting in a final sample of 87 participants (*M*_age_ = 29.23, *SD*_age_ = 9.55; 45.9% male). Participants were shown 10 agreeableness and 10 extraversion items (item order was random for each participant) taken from the BFI-44 (John & Srivastava, 1999) and were asked to indicate how much they would like someone who has the respective trait (e.g., is outgoing, sociable) on a 7-point scale, ranging from 1 (*would not like at all*) to 7 (*would like a lot*). Average ratings for each trait are shown in Supplemental Table S1. Although agreeableness items were associated with higher likability rating than extraversion items on average (mean difference = 1.88, *t*(84) = 18.56, *p* < .001), we selected three agreeableness and three extraversion items that reached similar average likability scores (agreeableness: *M* = 5.68, *SD* = 1.00; extraversion: *M* = 5.56, *SD* = 0.93; *t*(84) = .88, *p* = .381) and used them to develop the manipulation text for the main study.

### Main Study

#### Design and procedure

Similar to Study 1, we used a 2 (*Trustee Agreeableness*: high vs. low) × 2 (*Trustee Extraversion*: high vs. low) between-subjects design. In contrast to Study 1, we opted to avoid using deception and asked participants to imagine that they would be matched with another participant of this study in a decision-making task (the trust game). We used the same trust game instructions as in Study 1, with the exception that, this time, trustee’s decision was binary (i.e., they could choose to either keep half of the total amount and transfer the other half back to the trustor (each player gets £1.50); or to keep all of it (trustor gets nothing, trustee gets £3).

After checking participants’ understanding of the game rules (we used the same comprehension check question as in Study 1), participants were provided with additional information regarding their interaction partner. Similar to Study 1, participants read that Person 2’s name was Cory, and that Cory was either agreeable (or disagreeable) or extraverted (or introverted). Using three agreeableness and three extraversion BFI-44 items that achieved similar likability scores in the pretest, we constructed four profiles reflecting the different combinations of agreeableness and extraversion (see Table 2). We also varied whether the extraversion- versus agreeableness-related information was presented first. Trait order had no significant main or interaction effects on any of the dependent measures (all *p*s > .16), so for brevity reasons, we will present the analyses without this factor.

**Table 2.** Manipulation of Trustee Personality, Study 2.

	High extraversion	Low extraversion
High agreeableness	Cory is sociable and outgoing, is full of energy, and generates a lot of enthusiasm. Cory is helpful and unselfish with others, has a forgiving nature, and is never cold or aloof.	Cory is neither sociable nor outgoing, lacks energy, and does not generate much enthusiasm. Cory is helpful and unselfish with others, has a forgiving nature, and is never cold or aloof.
Low agreeableness	Cory is sociable and outgoing, is full of energy, and generates a lot of enthusiasm. Cory is unhelpful and selfish with others, does not have a forgiving nature, and can be cold and aloof.	Cory is neither sociable nor outgoing, lacks energy, and does not generate much enthusiasm. Cory is unhelpful and selfish with others, does not have a forgiving nature, and can be cold and aloof.

To measure *perceived trustworthiness*, participants indicated how likely Cory would be to keep half of the total amount and transfer the other half back to them, or keep all of the total amount, assuming they transfer Cory their £1 (1 = *Cory will definitely keep all of it*; 7 = *Cory will definitely transfer half to me*). To measure *perceived likability*, we used the interpersonal liking scale (Wojciszke et al., 2009). Participants rated Cory on the following three items: “I have warm feelings about Cory,” “I like Cory,” and “I feel close to Cory” (1 = *strongly disagree*, 7 = *strongly agree*; Cronbach’s α = .91). The order of perceived trustworthiness and likability was randomized across participants; as it had no significant main or interaction effects (all *p*s > .07), we did not include it in the main analyses.

Participants were then asked to indicate whether they would transfer their endowment (£1) to Cory or not (1 = *transfer*, 0 = *no transfer*). This constituted a measure of hypothetical behavioral trust and represented our dependent variable.

Finally, using a 5-point scale, participants completed a manipulation check by rating Cory on the dimensions of agreeableness and extraversion (we used the same items as in the manipulation check; Cronbach’s α = .95 and .83 for extraversion and agreeableness, respectively).

#### Participants

We aimed at collecting at least 200 participants to achieve 80% power to detect an effect of OR = 2.46 (α = .05). To compensate for participants failing the comprehension question, we recruited 300 participants on Prolific Academic. Individuals who participated in Study 1 or in the pretest were not invited to participate in this study. Of the 300 participants, 14 did not respond correctly to the comprehension question and were removed, resulting in a final sample of 287 individuals (*M*_age_ = 26.37, *SD*_age_= 7.42; 36.9% male).

### Results

#### Manipulation check

A 2 × 2 MANOVA (with perceived agreeableness and perceived extraversion as outcomes) showed that agreeable trustees were perceived as being more agreeable (*M* = 4.11, *SD* = 0.82) than disagreeable trustees (*M* = 1.80, *SD* = 0.83), *F*(1, 282) = 559.98, *p* < .001, $\eta_{p}^{2}$ = .67, 95% CI = [.62, .71], and that extraverted trustees were perceived as more extraverted (*M* = 4.21, *SD* = 0.90) than introverted trustees (*M* = 1.64, *SD* = 0.81), *F*(1, 282) = 643.49, *p* < .001, $\eta_{p}^{2}$ = .70, 95% CI = [.65, .73]. Trustee agreeableness did not significantly affect participants’ perception of trustee extraversion, *F*(1, 282) = 1.44, *p* = .231, and trustee extraversion did not significantly affect participants’ perception of trustee agreeableness, *F*(1, 282) = 0.91, *p* = .340. The interaction between trustee agreeableness and trustee extraversion was not significant, *F*(2, 282) = 0.65, *p* = .521. In addition, perceived extraversion was unrelated to perceived agreeableness (*r* = .11, *p* = .067).

#### Trust decisions

Following preregistered analysis plan, we used logistic regression with participants’ trust decision (1 = *trust*, 0 = *no trust*) as dependent variable. In Model 1, we regressed trust decision on the two manipulated factors—trustee extraversion and agreeableness—as predictors (*high* = 1, *low* = 0). Participants were more likely to trust agreeable than disagreeable trustees (OR = 12.93, *p* < .001, 95% CI = [7.21, 24.57]). In contrast, trustee extraversion did not predict higher trust (OR = 1.15, *p* = .628, 95% CI = [0.65, 2.04]); see Figure 1. In Model 2, we added an interaction between trustee agreeableness and extraversion, which was not significant (OR = 0.94, *p* = .929, 95% CI = [0.28, 3.26]).

#### Perceived trustworthiness and likability

**Figure 2.**
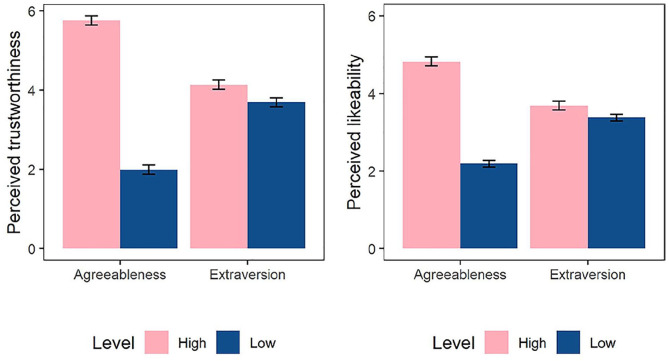
Perceived trustworthiness (left) and likability (right) as a function of target agreeableness and extraversion. *Note*. Error bars represent standard errors.

We regressed perceived trustworthiness and likability on target agreeableness and extraversion. Participants perceived both more agreeable (*b* = 3.76, *p* < .001, 95% CI = [3.45, 4.07], β = .81) and more extraverted (*b* = 0.37, *p* = .020, 95% CI = [0.06, 0.69], β = .08) targets as more trustworthy. Target agreeableness was associated with more liking (*b* = 2.63, *p* < .001, 95% CI = [2.34, 2.91], β = .73), whereas target extraversion was not (*b* = 0.26, *p* = .071, 95% CI = [−0.02, 0.55], β = .07). The interaction effects between target agreeableness and target extraversion were not significant for either of the outcomes (*p*s > .35). The average perceived trustworthiness and likability are shown in Figure 2.

**Figure 3.**
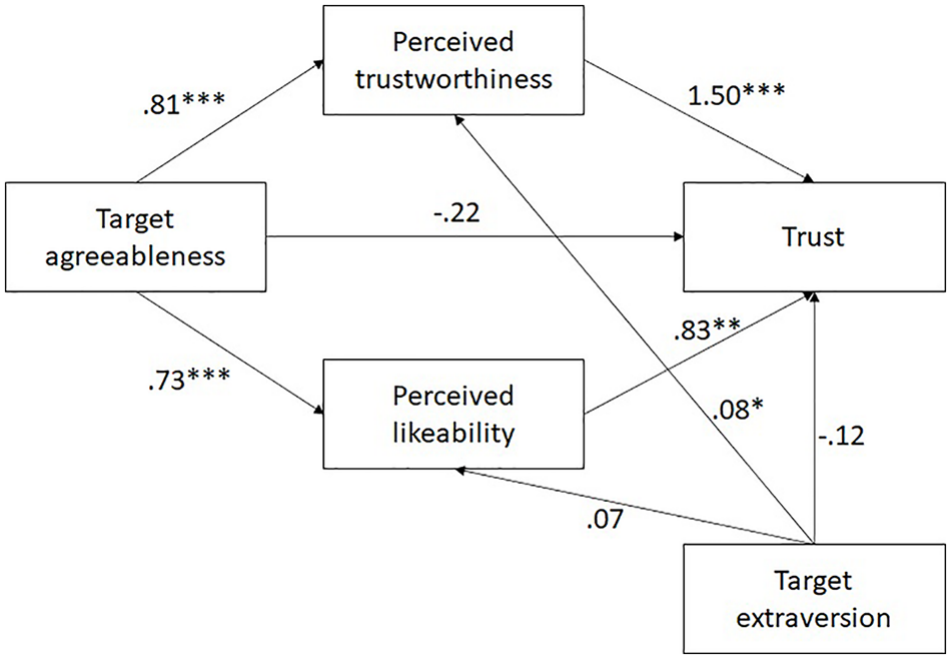
Parallel mediation, Study 2. *Note*. All variables (except for trust) have been standardized before the analyses and the respective path coefficients can be interpreted in terms of predicted change in standard deviations. The effects of perceived trustworthiness, likability, and target extraversion on trust are expressed in a log-odds metric. *p < .05. **p < .01. ***p < .001.

Next, we tested whether perceived trustworthiness mediated the effect of target agreeableness on trust above and beyond perceived liking, in a parallel mediation model. Following the preregistered analysis plan, we included target extraversion as a covariate. We used the Process macro V. 3.4 (Model 4; Hayes, 2017). The path coefficients are shown in Figure 3. Both indirect effects were significant: 1.21, 95% CI = [.69, 1.95], for perceived trustworthiness, and 0.61, 95% CI = [.20, 1.10], for liking. Hence, the effect of target agreeableness on trust cannot be explained solely by higher likability of agreeable targets.

As target extraversion did not significantly affect trust, we did not conduct the respective mediation analysis.

#### Exploratory (not preregistered) analyses

We additionally explored whether the effect of target agreeableness on trust is robust when controlling for perceived liking. We regressed trust on target agreeableness and perceived liking. Target agreeableness significantly predicted trust (OR = 2.89, *p* = .009, 95% CI = [1.31, 6.49]) above and beyond liking (OR = 2.05, *p* < .001, 95% CI = [1.56, 2.75]). Hence, the effect of target agreeableness on trust holds above and beyond just liking.

### Discussion

Study 2 replicated the results of Study 1, with a manipulation of extraversion that emphasized the aspects that arguably drive its likability (e.g., being outgoing and sociable). Nevertheless, similar to Study 1, people’s trust decisions were driven by target agreeableness rather than extraversion. Importantly, by additionally assessing perceived trustworthiness and likability, we showed that the effect of target agreeableness on trust cannot be simply explained by liking.

## Study 3

Studies 1 and 2 showed that people’s trust decisions are guided by their partner’s agreeableness (and not extraversion). In these studies, participants were provided with explicit information about their interaction partners’ personality. In real life, however, people are usually left to guess or make informed inferences about what others are like. Therefore, in Study 3, we asked, “When people have no explicit knowledge about their interaction partners’ personalities, do they still guide their decisions in social dilemma situations?” We explored whether people’s trust in their interaction partners in real-life social encounters is predicted by their interaction partners’ (actual) personality traits. We focused on real-life social dilemmas, such as workplace collaboration, and tested whether people are more likely to put trust in agreeable (but not extraverted) team members. Such collaborative projects, where individual group members decide how much effort to exert toward a common goal, are a canonical example of social dilemmas (Dawes, 1980). Although our focus was on agreeableness and extraversion, the study included the remaining Big Five traits (openness, conscientiousness, and neuroticism) as well.

Participants filled in the Big Five scales and, roughly a week later, joined a lab session where they worked together in small groups on a series of tasks and, at the end of the session, indicated their willingness to trust their group members, using a trust game paradigm. We used a round-robin design where each person in a group interacts and evaluates every other person in the same group (Kenny, 1994). This design allowed us to explore whether individuals’ self-reported personality scores predict their team members’ willingness to trust them.

This study was part of a larger project: it included measures that were collected for other research questions. The list of all the measures included can be accessed at the project’s Open Science Framework (OSF) page (https://osf.io/b68cs/).

### Method

#### Design and procedure

As participants signed up for the study, they were asked to complete an online questionnaire that included the Big Five scales and to sign up for a lab session to take place 1 to 2 weeks later. That way, there was always a time lag between filling in the initial personality scales and participating in a later lab session.

As part of the online questionnaire, participants completed the IPIP inventory (Goldberg, 1992) that included all Big Five scales measured with 10 items each. Responses were given on a 5-point scale, ranging from 1 (*very inaccurate*) to 5 (*very accurate*). All scales showed good reliabilities (alphas between .73 and .88).

Participants came to the lab in groups of three to six people, with an average of 5.58 (*SD* = 0.65) people. One group was handled per lab session. Most participants (90.2%) indicated that they did not know other members of their group before the lab session (either never met before or saw them on campus but never talked or just did not know them well). Participants took a seat at a group table in a way that they not only faced each other (which facilitated group work) but were also far enough from each other to allow a private completion of the questionnaire. Participants were given badges with their personal identifiers (“Participant A,” “Participant B,” and up to “Participant F”) and tablets that they used to read the instructions and answer the questions. After participants were seated, the experimenter asked them to put on the badges, launch the survey on their tablets, and follow the instructions in the survey. The experimenter then left the room.

Participants read that they would solve a number of tasks as a group. The instructions for each task were shown on their tablets. After everyone in the group read the instructions, participants were asked to discuss the task together, come up with a solution as a group, and write it down on a “Group Answer Sheet.” There was a certain amount of time (between 1 and 5 min) allocated to each task and participants were asked to try not to exceed this time. There were five tasks that involved three riddles (taken from Arieli & Sagiv, 2018) and two unusual uses tasks (Guilford, 1967). Overall, participants spent about 20 min working on the tasks.

After the group work was over, participants were asked to return to their individual tablets and answer a couple of questions about the tasks they had completed and their groupmates. Participants were introduced to a trust game. In this version of the game, both players started with €5 each. Person 1 could choose to transfer their €5 to Person 2 or not. If Person 1 transferred €5 then it was tripled and given to Person 2 (so Person 2 has €20). Person 2 could then choose how much they wanted to transfer back to Person 1 (between €0 and €15). The instructions were followed by an understanding check question: What happens if Person 1 transfers €5 and Person 2 transfers back €10? (“Person 1 earns €5, Person 2 earns €10”; “Person 1 earns €10, Person 2 earns €10”; “Person 1 earns €0, Person 2 earns €20”). Afterward, participants were assigned to the role of Person 1 and were asked to rank order the other members of their group, depending on how likely they would be to transfer them their €5 (1 = *most likely to transfer the money to*, 2 = *less likely to transfer the money to*, until all members of the group were ranked). Thus, depending on the number of people in their respective group, this number ranged between 1 and 3, 1 and 4, 1 and 5, and 1 and 6. We decided to use the ranking method to make sure that there is enough variance and no ceiling effects. For the analyses, we recoded the trust measure, such that higher values reflect a higher level of trust, and group-mean centered it to adjust for different group sizes.

#### Participants

The study was advertised to first-year undergraduate psychology students at a Dutch university. Participants were compensated with course credits. The hour-long lab sessions were scheduled for every workday for 2 weeks. Given the size of the subject pool and the lab capacity, we expected to recruit about 300 participants. Overall, 360 individuals completed the online questionnaire. Eight failed an attention check question (“To monitor data quality, please select the middle of the scale here”) and were removed. Of the remaining participants, 309 participated in a lab session, of whom 67 failed to correctly answer the question checking their comprehension of the trust game’s rules, 34 had missing values on the key variables (trust, agreeableness, or extraversion), and two entered the wrong person ID, resulting in a final sample of 206 (*M*_age_ = 19.67, *SD*_age_ = 1.89; 88% female). This sample size gave us an 80% power to detect relatively small (|*r*| = .19, α = .05, two-tailed) effects (e.g., association between personality and being trusted).

### Results

#### Descriptive statistics and zero-order correlations

Participants’ agreeableness was related to a higher likelihood of being trusted (*r* = .15, *p* = .034, 95% CI = [.01; .28]). Neither participants’ extraversion nor any other Big Five trait was associated with being trusted (all *p*s > .22; see Supplemental Table S2).

#### Social relations model (SRM)

SRM is a standard method for analyzing round-robin data. The model determines how much variance in participants’ trust to each other can be explained by the characteristics of the group they are in (group effects), the target (target effects), the perceiver (perceiver effects), and unique combinations of perceiver and target characteristics (relationship effects; Kenny et al., 2006).

We used multilevel modeling, as implemented in the SRM_R app (Kenny & Wong, 2016), based on the approach suggested by Snijders and Kenny (1999) and the code written by Knight and Humphrey (2019) for the nonlinear mixed-effects (nlme) package in R. We relied on this method as this is the only currently available method that can handle groups with less than four members. The SRM model is a type of crossed random effects model with partially correlated random factors. The model included random effects of the group, the perceiver, the target, and the dyad (unique perceiver–target combinations). Following Knight and Humphrey (2019), the covariances among the random effects were fixed to zero, except for the covariance between the random perceiver and target effects (generalized reciprocity) and the covariance between the relationship effects for the members of the same dyad (dyadic reciprocity). The results of these analyses are shown in Table 3. All predictor and outcome variables were standardized before the analyses, meaning that the coefficients can be interpreted as standardized effect sizes.

**Table 3.** Social Relations Model Results, Study 3.

	DV: trust, *b*			
	Model 0	Model 1	Model 2	Model 3
Fixed effects				
Target characteristics				
Agreeableness	—	.09^†^	—	.10*
Extraversion	—	—	.01	.01
Openness	—	—	—	−.02
Conscientiousness	—	—	—	−.07
Emotional stability	—	—	—	−.06
Perceiver characteristics				
Agreeableness	—	—	—	.06
Extraversion	—	—	—	−.01
Openness	—	—	—	−.01
Conscientiousness	—	—	—	.01
Emotional stability	—	—	—	−.003
Random effects				
Team	.000	.000	.000	.000
Perceiver	.002	.001	.002	.002
Target	.147***	.140***	.149***	.134***
Dyad^a^	.852	.853	.852	.861
Generalized reciprocity	.012	.010	.012	.010
Dyadic reciprocity	.158**	.155**	.158**	.158**

*Note*. Fixed effects are standardized regression coefficients (obtained by standardizing all predictor and outcome variables; SRM_R app does not provide confidence intervals); random effects can be interpreted as variance attributed to the respective factors. DV = dependent variable; SRM = social relations model.

a is confounded with error variance and the software provides no significance test for it.

† *p* < .10. *p < .05. **p < .01. ***p < .001.

A null model (that included only the random effects but no predictors (Model 0 in Table 2) showed that a significant share of variance in being trusted could be attributed to target characteristics (14.7%, *p* < .001), whereas only very little variance could be explained by perceiver characteristics (0.2%, *p* = .62). This is not surprising as perceivers were asked to rank all members of their group, that is, all perceivers had to use values from 1 to 5 (in a group with six members), reducing perceiver variance. Within each dyad, individuals who assigned a higher trust rank to a particular group member were more likely to be assigned a higher trust rank by this member (dyadic reciprocity: .16, *p* = .002).

Model 1 included target agreeableness as a predictor. More agreeable targets were slightly more likely to be trusted than less agreeable targets although this effect was short of the conventional level of significance (β = .09, *p* = .055). Model 2 tested the effect of target extraversion and showed that it was not associated with being trusted (β = .01, *p* = .78). In Model 3, we included all Big Five scores of both targets and perceivers: of all the Big Five traits, only target agreeableness predicted being trusted (β = .10, *p* = .037).

#### Exploratory analyses

In addition to the trust decision, participants rated each other on the dimensions of morality (e.g., “To what extent is the [target] trustworthy?”), competence (e.g., “To what extent is the [target] competent?”), performance (e.g., “How well did the [target] perform on the group tasks?”), and respectful behavior (e.g., “The [target] treated other members with respect?”). A set of further analyses explored the effect of target personality on these other dimensions. We provide a brief summary of the results here and refer the reader to the supplemental materials for details. We used SRM models (with the same specification as in the main analyses) to explore the effects of target agreeableness and extraversion on perceived morality, competence, performance, and respectful behavior. Participants perceived extraverted groupmates as being more competent (β = .10, *p* = .004) and performing better (β = .22, *p* < .001) than less extraverted groupmates; Participants perceived more (vs. less) agreeable groupmates as more moral (β = .10, *p* = .006) and competent (β = .09, *p* = .026; although the competence effect disappeared after controlling for target extraversion). No other effects reached significance. Next, we explored whether attribution of morality mediated the effect of target agreeableness on being trusted. We estimated the association between target agreeableness and perceived morality (path “a”) and perceived morality and trust (path “b”) in separate SRM models and used Monte Carlo simulations to determine the significance of the indirect effect (a*b; Selig & Preacher, 2008). The indirect effect was significant (.03, 95% CI = [.006, 06]), providing support for the mediation.

## Discussion

Study 3 extended the findings of Studies 1 and 2 to real-life social dilemmas: group work. Similar to social dilemmas, group work creates a situation where individuals might obtain benefits by acting selfishly (e.g., not investing effort but claiming the rewards). After working together for just about 20 min, individuals were more willing to entrust their monetary endowment to more (vs. less) agreeable team members. Consistent with the results of Studies 1 and 2, extraverts were not considered more trustworthy than introverts. Taking into consideration the relatively high *p* values, the absence of preregistration, and the fact that the study was powered for a larger effect (*r* = .19, as aforementioned) than the one we detected (*r* = .15, as aforementioned), these findings provide suggestive evidence that even when people have no explicit knowledge about their interaction partners’ agreeableness, it seems to guide their decisions in social dilemma situations.

## Study 4

Studies 1 to 3 showed that partner agreeableness (but not extraversion) guides individuals’ trust. Are people aware of how their personality shapes other people’s trust decisions and do they use this knowledge strategically? In Study 4, we examined whether people attempt to appear more agreeable when trying to gain others’ trust. Participants were asked to take on the role of trustees and to fill in personality scales, with the understanding that their scores would remain private (private condition) or that they would be shown to trustors to help them make the decision (public condition). We expected that participants would try to appear more agreeable and, given the social desirability of extraversion, also more extraverted in the public (vs. private) condition.

Measures, data collection, and analyses were preregistered (https://aspredicted.org/fm7ia.pdf). There were no deviations from the preregistration.

### Method

#### Design and procedure

Participants learned that they would interact with another participant of the study. They were shown the instructions of the trust game, the same as in Study 1. However, this time, as we were only interested in trustees’ responses to personality measures, all participants were assigned the role of Person 2, the trustee (note that the words “trust,” “trust game,” “trustor,” or “trustee” were not used in either of the studies). We paid out 10 randomly selected participants, based on their decision as trustee (assuming that the trustor decided to send them the money, to ensure a fair treatment despite deception). After making themselves familiar with the payoff structure, participants responded to a question checking their understanding of the rules of the game (the same as in Study 1).

Afterward, participants were randomly assigned to one of two conditions (public or private). In both conditions, they filled in the extraversion (10 items, sample item “Don’t mind being the center of attention,” α = .90) and agreeableness (10 items, sample item “Sympathize with others’ feelings,” α = .82) scales from the IPIP (Goldberg, 1992). A 7-point response scale ranging from 1 (*very inaccurate*) to 7 (*very accurate*) was used.

In the public condition, before filling out the extraversion and agreeableness scales, participants were told that their answers would be shown to their interaction partner (Player 1) who will use this information to make the decision to transfer them the money or not. In the private condition, participants were just asked to answer the questions about themselves as accurately as they could. The complete manipulation text is provided in the supplemental materials. We counterbalanced the order in which the agreeableness versus the extraversion scales were presented.^3^ Participants’ agreeableness and extraversion scores constituted our dependent variables.

After filling in the scales, participants read that Person 1 (the trustor) had made their decision. All participants learned that Person 1 decided to transfer them £1 and that they had £3 now. They were asked to indicate how much they would like to transfer back to Person 1 (from £0 to £3). We used participants’ responses to this question as an indicator of their trustworthiness (we did not have any predictions regarding this measure; rather, we included it just to make the study’s cover story more convincing). Finally, participants filled in sociodemographic information. Similar to Study 1, 10 randomly selected participants were paid out.

#### Participants

We recruited participants on Prolific Academic. To be able to detect a small effect (*d* = .35, independent-sample *t* test; α = .05, two-tailed test, 80% power), we aimed at collecting at least 260 responses. To compensate for participants failing the comprehension question of the trust game, we decided to recruit 300 participants. Out of 301 individuals who completed the survey, 43 failed the question testing whether they understood the instructions of the trust game, resulting in a final sample of 258 (*M*_age_ = 28.90, *SD*_age_ = 10.65; 51.2% male) individuals.

### Results

#### Effect of the experimental condition on extraversion and agreeableness

**Figure 4.**
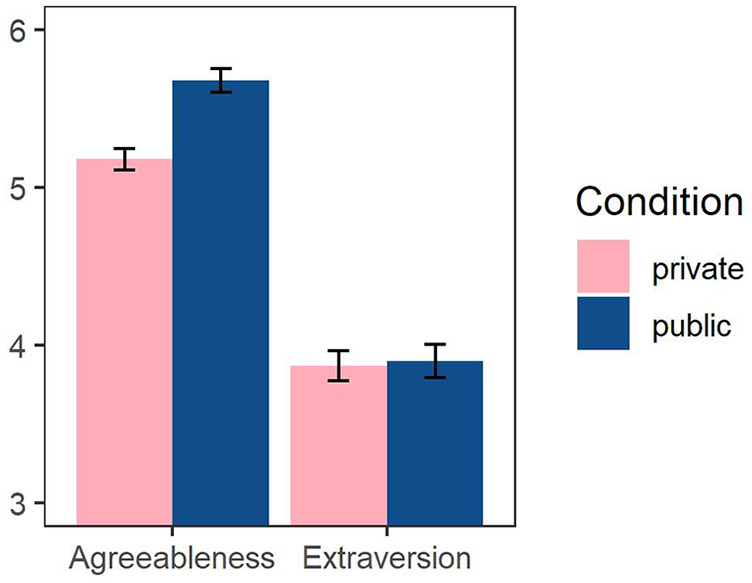
Extraversion and agreeableness scores by condition, Study 4. *Note*. Error bars represent standard errors.

Participants reported being more agreeable in the public (*M* = 5.68, *SD* = 0.86) than in the private condition (*M* = 5.18, *SD* = 0.79), *t*(256) = 4.83, *p* < .001, *d* = 0.60, 95% CI = [.24, .95]. However, the experimental condition had no significant effect on participants’ self-reported extraversion scores, *t*(256) = 0.21, *p* = .83; see Figure 4. We also tested the effect of the experimental condition on agreeableness, when controlling for extraversion, and the effect of the experimental condition on extraversion, when controlling for agreeableness (using analysis of covariance [ANCOVA]). The effect of the condition on agreeableness remained significant (*p* < .001) and the effect of the condition on extraversion remained not significant (*p* = .375).

In addition to the preregistered analyses reported above, to test whether the effect of the experimental condition on agreeableness is significantly different from its (null) effect on extraversion, we ran a 2 (condition) × 2 (trait) mixed analysis of variance (ANOVA). This analysis yielded a significant interaction effect, *F*(1, 256) = 8.92, *p* = .003, $\eta_{p}^{2}$ = .034, 95% CI = [.004, .09], that is, knowing that their scores will be shown to the trustors made trustees overreport their agreeableness, but not their extraversion.

#### Trustworthiness

In the exploratory (not preregistered) part of the analyses, we examined whether the experimental condition (public vs. private), together with participants’ self-reported agreeableness and extraversion scores, predicted participants’ trustworthiness (i.e., how much money they transferred back). An independent-sample *t* test showed that participants in the public condition appeared to transfer slightly more money (*M* = 1.58 out of £3, *SD* = 0.51) than participants in the private condition (*M* = 1.47 out of £3, *SD* = 0.45) although we are hesitant to interpret this effect as it did not reach the conventional level of significance, *t*(255) = 1.84, *p* = .067, *d* = .23, 95% CI = [−.02, .48]. Neither agreeableness nor extraversion were significantly associated with trustworthiness (agreeableness: *r* = .077, *p* = .22, 95% CI = [−.05, .20]; extraversion: *r* = .01, *p* = .88, 95% CI = [−.11, .13]) and this was the same across the conditions (Condition × Extraversion and condition × Agreeableness interactions: *p*s > .20; however, note that the correlation between agreeableness and trustworthiness in the private [i.e., control] condition was closer to the findings usually reported in the literature: *r* = .13, *p* = .14, 95% CI = [−.05, .30]).

### Discussion

When faced with the dilemma of trust, people have a preference for agreeable (but not extraverted) others (Studies 1–3). In Study 4, we showed that this preference might have consequences for peoples’ impression management strategies: People try to appear more agreeable when they have to convince others to trust them. Importantly, although higher levels on both agreeableness and extraversion are more socially desirable than lower levels (John & Robins, 1993), participants overreported agreeableness but not extraversion. Hence, individuals’ attempt to appear more agreeable is likely to be explained by their belief about the importance of (specifically) agreeableness in social dilemmas, rather than by their general impression management goals. Still, this finding could be partially driven by individuals’ tendency to overreport agreeableness in public contexts more generally. Hence, future studies need to compare individuals’ tendency to overreport agreeableness across different public contexts: the ones where they need to build trust versus not.

## General Discussion

When interacting with strangers, which personality inferences guide the development of trust? Drawing from existing research on social perception (Abele et al., 2016; Imhoff & Koch, 2017), we examined the role of two traits from the Big Five framework: extraversion and agreeableness. On one hand, previous work has emphasized the social benefits of extraversion (Anderson et al., 2001; Judge et al., 2002): extraverted individuals have larger networks, are liked by their colleagues and bosses, attain status easily, and enjoy the multiple benefits of a modern society that is often seen as geared toward extraverts (Cain, 2012). On the other hand, social perception research has shown that characteristics usually associated with extraversion, such as sociability and agency, do not necessarily predict positive social impressions (Hartley et al., 2016; Landy et al., 2016). Hence, despite their general popularity (Anderson et al., 2001; Ilmarinen et al., 2015), extraverts might not have a reputational advantage in social dilemmas of trust. Indeed, in different situations, such as incentivized trust games played online with strangers (Study 1) and laboratory observation of work groups (Study 3), we showed that people do not trust extraverts more than introverts. These findings add to a literature that has started to reveal the potential downsides of extraversion, such as long-term status losses in interdependent teams (Bendersky & Shah, 2013) and extraverted leaders’ negative effect on proactive employees (Grant et al., 2011).

In contrast to extraversion, our results suggest that people consider agreeableness to be important in social dilemma situations; people trust more (vs. less) agreeable individuals. Participants awarded more trust to agreeable (and not extraverted) others even when the manipulations of agreeableness and extraversion were matched to yield a similar level of liking; Furthermore, the effect of partner agreeableness on trust emerged above and beyond its effect on liking (Study 2). Interestingly, partner agreeableness affected individuals’ trust decisions even when they did not have any explicit knowledge about their partner’s standing on the agreeableness scale (Study 3). Specifically, participants spent about 20 min working together, which turned out to be enough for them to decide whom of their group members they trusted most and least. Replicating the results of Studies 1 and 2, participants were more willing to entrust a monetary endowment to more (vs. less) agreeable team members, although they had no explicit knowledge about their team members’ personality (which was obtained using self-report scales days or even weeks before the participants entered the lab). However, given that the respective *p* values were close to .05, the study was not preregistered, and could have lacked power (as aforementioned), further replications are needed to be confident in these results.

We also demonstrated that people are aware of others’ preferences for agreeable partners and that this awareness has consequences for peoples’ impression management strategies. Specifically, in Study 4, participants overreported their level of agreeableness (but were honest about their extraversion) when their personality scores would be shared with potential interaction partners. Hence, people are more likely to trust agreeable (but not extraverted) others and, when they have to convince others to trust them, people try to appear more agreeable (but not more extraverted). Taken together, these findings suggest that people might be quite good at predicting what will make them appear trustworthy.

### Future Research Directions

We have shown that individuals trust more (vs. less) agreeable partners even in the absence of explicit knowledge about their partners’ actual level of agreeableness. Yet it remains unclear whether, to make their trust decisions, individuals rely on specific “agreeableness cues” and what these “agreeableness cues” might be. Previous research has shown that people’s judgment of others’ agreeableness based on “thin slices” of their behaviors, such as silent videos, photographs, or social media profiles, is more accurate than chance (Connelly & Ones, 2010; Funder & Sneed, 1993; Tskhay & Rule, 2014). This accuracy has been explained by individuals’ personality traits being reflected in their verbal and nonverbal behavior, facial expressions, or looks (Gosling et al., 2002; Naumann et al., 2009). For example, agreeable people are more likely to have a friendly/smiling profile picture on social media (Hall et al., 2014) and to create avatars that others would want to befriend (Fong & Mar, 2015); in their everyday conversations, agreeable people are less likely to swear and are more likely to express positive emotions (Mehl et al., 2006; Yarkoni, 2010). Hence, spending 20 min working together could have allowed individuals to pick up on such cues and use them to make trust decisions. Future studies could be conducted to examine what specific cues individuals rely on, whether these cues provide a valid signal of agreeableness and/or trustworthiness, and whether individuals’ judgment of their teammates’ trustworthiness is accurate.

There is also a need to determine whether the observed effects of agreeableness are driven by some of its facets (e.g., altruism) but not others (e.g., straightforwardness). In addition, the partner-level effects of agreeableness could be different when operationalized using a six-factor model of personality, namely, honesty–humility (H), emotionality (E), extraversion (X), agreeableness (A), conscientiousness (C), and openness to experience (O) (HEXACO) that considers forgiveness and temper-control as a defining feature of agreeableness, leaving selfishness and altruism to describe a different trait: honesty–humility (Ashton et al., 2014). Interestingly, studies on the associations between HEXACO traits and trustworthy behaviors in trust games point at honesty–humility (but not agreeableness) as a consistent predictor of trustworthiness (Thielmann & Hilbig, 2015). We hope that future studies will reveal which specific facets and conceptualizations of agreeableness most strongly predict being trusted.

These findings add to several research areas. First, they contribute to the literature on the role of personality in trust at zero-acquaintance. Most previous research in this area has focused on actor-level effects of personality by exploring how an individual’s personality affects that individual’s trust decisions (Thielmann et al., 2020). In contrast, these studies suggest that personality does not only have actor- but also partner-level effects. In other words, it is not only the personality of the trustor but also the personality of the trustee that matters. For example, the study of work groups (Study 3) showed that a significant share of variance in trust could be attributed to differences between the trustees. Comparing the importance of trustor versus trustee characteristics directly and exploring whether the importance of partner-level effects of personality extends to further social dilemmas (e.g., cooperation) could be interesting avenues for future studies.

Second, our findings contribute to the literature on the role of impression management in personality assessment. We demonstrated that people are not only aware of others’ preferences for agreeableness but also let this awareness shape their individual responses to the agreeableness scale. Specifically, knowing that their agreeableness scores will be shown to their interaction partners made participants report higher agreeableness (Study 4). Although it remains to be explored whether this effect is specific to the dilemmas of trust or extends to other impression management situations, these findings might have implications for the ongoing debate regarding whether social desirability motives might threaten the validity of personality assessment as a personnel selection method (Cao & Drasgow, 2019; Griffith et al., 2016). Relatedly, future research should assess whether people’s knowledge that it is important to appear agreeable in trust situations would also cause them to *behave* in a more agreeable way.

Finally, our results suggest that, although extraverts might be popular (e.g., Jensen-Campbell et al., 2002; Lönnqvist & große Deters, 2016), they do not necessarily elicit a higher level of trust from others, and thus offer a more nuanced view of the social consequences of extraversion.

## Supplemental Material

sj-docx-1-psp-10.1177_01461672221086768 – Supplemental material for The Effects of Partner Extraversion and Agreeableness on Trust Click here for additional data file. Supplemental material, sj-docx-1-psp-10.1177_01461672221086768 for The Effects of Partner Extraversion and Agreeableness on Trust by Olga Stavrova, Anthony M. Evans and Ilja van Beest in Personality and Social Psychology Bulletin

## Notes

1. We wanted to explore whether some combinations of agreeableness and extraversion might be seen as more coherent or “natural” than others and whether this might further affect trust. That was not the case. The results are summarized in the supplemental materials.

2. In the preregistration, we mistakenly stated that this sample size would allow us to detect odds ratio (OR) = 1.7 (instead of OR = 2.45) because we did not correct for the predictor following a binomial distribution.

3. Filling in the agreeableness (vs. extraversion) scale first resulted in slightly higher agreeableness scores, *F*(1, 254) = 9.47, *p* = .002; however, this effect did not statistically differ across the conditions, *F*(1, 254) = 0.50, *p* = .48.
