# Supplementary material for: The Effects of Partner Extraversion and Agreeableness on Trust
Source: Pers Soc Psychol Bull. 2022 Apr 28;49(7):1028–42. doi: 10.1177/01461672221086768 (PMC10302358; doi:10.1177/01461672221086768)
Supplement: sj-docx-1-psp-10.1177_01461672221086768 – Supplemental material for The Effects of Partner Extraversion and Agreeableness on Trust [file sj-docx-1-psp-10.1177_01461672221086768.docx]

**Supplementary materials**

**Study 1**

For exploratory reasons (we wanted to explore whether some combinations of agreeableness and extraversion might be seen as more coherent or “natural” than others and whether this might further affect trust), we also asked participants to indicate to what extent they thought that Cory had “a coherent personality” and “a contradictory personality” (reverse-coded). Their responses to these two questions were combined into a measure of “perceived personality coherence” (*r* = .30, *p* < .001).

Agreeable trustees were seen as having a more coherent personality (*r* = .33, *p* < .001). Neither extraversion, nor agreeableness x extraversion interaction affected perceived personality coherence (all *p*s > .40). Perceived personality coherence was positively related to trust (*r* = .21, *p* = .001), but when entered together with trustee agreeableness, only the latter predicted trust (agreeableness: OR = 4.01, *p* < .001; coherence: OR = 1.40, *p* = .063).

**Study 2**

Table S1

*Likeability ratings for each agreeableness and extraversion item from the pre-test*

|  | Average |
| --- | --- |
| *Agreeableness items* |  |
| Tends to find fault with others^#^ | 5.81 |
| **Is helpful and unselfish with others** | **5.95** |
| Starts quarrels with others^#^ | 6.17 |
| **Has a forgiving nature** | **5.78** |
| **Can be cold and aloof**^#^ | **5.31** |
| Is considerate and kind to almost everyone | 6.12 |
| Is sometimes rude to others | 5.84 |
| Is generally trusting | 5.83 |
| Likes to cooperate with others | 6.02 |
| *Extraversion items* |  |
| Is talkative | 4.94 |
| Is reserved^#^ | 3.44 |
| **Is full of energy** | **5.43** |
| **Generates a lot of enthusiasm** | **5.65** |
| Has an assertive personality | 4.88 |
| Is sometimes shy, inhibited^#^ | 3.44 |
| **Is outgoing, sociable** | **5.64** |
| Tends to be quiet^#^ | 3.83 |

*Note.* Negative items (denoted with ^#^) were reverse-coded. Items in bold were selected for the manipulation.

**Study 3**

**Exploratory analysis: Perceptions of morality, competence, performance and respectful behavior**

**Method**

After the group work was over, participants were asked to return to their individual tablets and answer a couple of questions about the tasks they had completed and their group mates. First, participants were asked to indicate to what extent they perceived each of their group members as honest, well-intentioned and trustworthy (averaged into a measure of *perceived morality;* α’s between .87 and .89), and competent, intelligent and efficient (averaged into a measure of *perceived competence;* α’s between .83 and .90). A 7-point scale ranging from 1 (not at all) to 7 (very much) was used. Next, participants rated each other’s behavior during the group work using the following questions: “How well did Participant A (vs. B … vs. F) perform on the group tasks (1 = very poorly, 10 = excellent)?”, “Did Participant A (vs. B … vs. F) participate in brainstorming sessions (1 = not at all, 5 = very)?”, “Did Participant A (vs. B … vs. F) propose problem-solving suggestions (1 = not at all, 5 = very)?”, “How much did Participant A (vs. B … vs. F) contribute to solving the group tasks (1 = very low, 7 = very high)?”, “Did Participant A (vs. B … vs. F) share his / her ideas and thoughts during group discussions? (1 = not at all, 5 = very)”, and “Participant A (vs. B … vs. F) put a lot of effort in the group work (1 = very inaccurately, 7 = very accurately)”. Participants’ responses to these questions were standardized and averaged into a measure of *perceived performance* (α’s between .90 and .94). Finally, participants indicated how accurately the following four statements describe each group member’s behavior during group work: “Participant A (vs. B … vs. F) treated other members with respect”, “Participant A (vs. B … vs. F) paid little attention to other members' statements”, “Participant A (vs. B … vs. F) talked down to others”, “Participant A (vs. B … vs. F) was friendly to other members of the group” (1 = very inaccurately, 7 = very accurately). After reverse-coding the negative items, we averaged them into a measure of *perceived respectful behavior* (α’s between .52 and .60).

**Results**

Zero-order correlations among the variables are shown in Table S1; the results of the Social Relations Model are shown in Table S2.

We conducted the SRM analyses using the same model specifications as in the analysis of trust reported in the main text. All predictor and outcome variables were standardized before the analyses, such that the coefficients can be interpreted as standardized effect sizes.

*Perceived morality.* Variance in morality ratings was attributed to both perceiver (45.3%, *p* < .001) and target (7.4%, *p* < .001) effects. Agreeable targets were rated more moral than less agreeable targets (β = .10, *p* = .006). Target extraversion was unrelated to perceived morality (*p* > .80).

*Perceived competence.* 32.3% (*p* < .001) of variance in competence perception was attributed to perceivers and 17.3% (*p* < .001) to targets. When considered independently of each other, both target agreeableness (β = .10, *p* = .026) and target extraversion *(*β = .12, *p* = .004) predicted perceived competence. However, when we entered all the Big Five traits of both targets and perceivers, only target extraversion remained significant (β = .11, *p* = .048). Among perceiver characteristics, agreeableness was associated with higher competence ratings (β = .16, *p* = .006) and openness with lower competence ratings (β = -.12, *p* = .028).

*Perceived performance.* 11.2% (*p* < .001) of variance in competence perception was attributed to perceivers and 53.1% (*p* < .001) to targets. Target agreeableness was unrelated to perceived performance (β = .06, *p* = .35), while target extraversion was: extraverted team members were more likely to be seen as high performers than their more introverted counterparts (β = .23, *p* < .001). Unexpectedly, more (vs. less) conscientious targets received worse performance ratings (β = -.14, *p* = .011). We speculate that this might be an artifact of the creative nature of the group tasks used; indeed higher levels of conscientiousness were associated with worse creative performance in previous research (Wolfradt & Pretz, 2001). None of the perceiver traits was significantly related to perceived performance (all *p*s > .16).

*Perceived respectful behavior.* 48.4% (*p* < .001) of variance in the perception of respectful behavior towards others was attributed to perceivers and 1.9% (*p* = .377) to targets. None of the target or perceiver traits were associated with the perception of target being respectful of others (all *p*s > .08).

*Exploratory mediation analysis.* Of perceived morality, competence, performance and respectful behavior, only morality was associated with target agreeableness. Therefore, we explored whether perceived morality mediated the effect of target agreeableness on being trusted. We estimated the association between target agreeableness and perceived morality (path “a”) and perceived morality and trust (path “b”) in separate SRM models (using the same specification as in the main analyses). The models included all other target and perceiver traits as covariates. We then used Monte Carlo simulations to determine the significance of the indirect effect (a*b) (Selig & Preacher, 2008). The indirect effect was significant (.03, 95% CI [.006, 06]), providing support for the mediation. The path coefficients are shown in Figure S1.

Table S2

*Means, standard deviations and zero-order correlations, Study 3*

| Variable | *M* | *SD* | 1 | 2 | 3 | 4 | 5 | 6 | 7 | 8 | 9 |
| --- | --- | --- | --- | --- | --- | --- | --- | --- | --- | --- | --- |
| 1. Trust^a^ | 0.05 | 0.86 |  |  |  |  |  |  |  |  |  |
| 2. Perceived morality^a^ | 6.00 | 0.50 | .35** |  |  |  |  |  |  |  |  |
|  |  |  | [.23, .47] |  |  |  |  |  |  |  |  |
| 3. Perceived competence^a^ | 5.75 | 0.59 | .33** | .57** |  |  |  |  |  |  |  |
|  |  |  | [.20, .45] | [.47, .66] |  |  |  |  |  |  |  |
| 4. Perceived performance^a^ | 0.01 | 0.71 | .43** | .37** | .71** |  |  |  |  |  |  |
|  |  |  | [.32, .54] | [.25, .48] | [.64, .78] |  |  |  |  |  |  |
| 5. Perceived respectful behavior^a^ | 4.24 | 0.44 | .12 | .40** | .35** | .23** |  |  |  |  |  |
|  |  |  | [-.01, .26] | [.28, .51] | [.23, .47] | [.09, .35] |  |  |  |  |  |
| 6. Target openness | 3.58 | 0.54 | -.02 | .02 | .11 | .16* | -.02 |  |  |  |  |
|  |  |  | [-.16, .12] | [-.12, .15] | [-.02, .25] | [.03, .29] | [-.16, .12] |  |  |  |  |
| 7. Target conscientiousness | 3.43 | 0.60 | -.03 | .09 | .01 | -.14* | -.01 | .15* |  |  |  |
|  |  |  | [-.16, .11] | [-.05, .22] | [-.13, .15] | [-.28, -.01] | [-.15, .13] | [.02, .28] |  |  |  |
| 8. Target extraversion | 3.11 | 0.71 | -.00 | -.03 | .10 | .29** | -.04 | .27** | .00 |  |  |
|  |  |  | [-.14, .14] | [-.16, .11] | [-.04, .23] | [.16, .41] | [-.17, .10] | [.14, .39] | [-.14, .14] |  |  |
| 9. Target agreeableness | 4.08 | 0.45 | .15* | .18* | .16* | .14 | .10 | .20** | .20** | .20** |  |
|  |  |  | [.01, .28] | [.04, .31] | [.02, .29] | [-.00, .27] | [-.04, .23] | [.07, .33] | [.06, .32] | [.07, .33] |  |
| 10. Target emotional stability | 2.87 | 0.70 | -.08 | .03 | .02 | .00 | .07 | -.00 | .14 | .25** | .00 |
|  |  |  | [-.22, .05] | [-.11, .16] | [-.11, .16] | [-.13, .14] | [-.07, .20] | [-.14, .14] | [-.00, .27] | [.12, .37] | [-.13, .14] |

*Note.* ^+^*p* < .10, ^*^*p* < .05, ^**^*p* < .01, ^***^*p* < .001; ^a^Average values computed for each target. Numbers in the brackets are 95% confidence intervals.

Table S3

*Social Relations Model results, Study 3*

|  | **DV: perceived morality,** β | | | |
| --- | --- | --- | --- | --- |
|  | Model 0 | Model 1 | Model 2 | Model 3 |
| **Fixed effects** |  |  |  |  |
| Target characteristics |  |  |  |  |
| Agreeableness | - | .10^**^ | - | .10^*^ |
| Extraversion | - | - | -.01 | -.03 |
| Openness | - | - | - | .04 |
| Conscientiousness | - | - | - | -.02 |
| Emotional stability | - | - | - | -.002 |
| Perceiver characteristics |  |  |  |  |
| Agreeableness | - | - | - | .11^+^ |
| Extraversion | - | - | - | -.04 |
| Openness | - | - | - | .01 |
| Conscientiousness | - | - | - | -.07 |
| Emotional stability | - | - | - | .11^+^ |
| **Random effects** |  |  |  |  |
| Team | .063 | .063 | .063 | .062 |
| Perceiver | .453^***^ | .452^***^ | .453^***^ | .448^***^ |
| Target | .074^***^ | .068^***^ | .075^***^ | .069^***^ |
| Dyad | .396 | .395 | .396 | .396 |
| Generalized reciprocity | -.011 | -.020 | -.010 | -.022 |
| Dyadic reciprocity | .016 | .016 | .016 | .016 |
|  | **DV: perceived competence,** β | | | |
|  | Model 0 | Model 1 | Model 2 | Model 3 |
| **Fixed effects** |  |  |  |  |
| Target characteristics |  |  |  |  |
| Agreeableness | - | .10^*^ | - | .05 |
| Extraversion | - | - | .12^**^ | .09^*^ |
| Openness | - | - | - | .06 |
| Conscientiousness | - | - | - | -.03 |
| Emotional stability | - | - | - | -.01 |
| Perceiver characteristics |  |  |  |  |
| Agreeableness | - | - | - | .16^**^ |
| Extraversion | - | - | - | .05 |
| Openness | - | - | - | -.12^*^ |
| Conscientiousness | - | - | - | -.01 |
| Emotional stability | - | - | - | .09^+^ |
| **Random effects** |  |  |  |  |
| Team | .057 | .049 | .073 | .057 |
| Perceiver | .323^***^ | .323^***^ | .323^***^ | .323^***^ |
| Target | .173^***^ | .171^***^ | .153^***^ | .173^***^ |
| Dyad | .442 | .442 | .444 | .442 |
| Generalized reciprocity | -.005 | -.016 | -.018 | -.005 |
| Dyadic reciprocity | -.045 | -.045 | -.044 | -.045 |
|  | **DV: perceived performance,** β | | | |
|  | Model 0 | Model 1 | Model 2 | Model 3 |
| **Fixed effects** |  |  |  |  |
| Target characteristics |  |  |  |  |
| Agreeableness | - | .10^+^ | - | .06 |
| Extraversion | - | - | .25^***^ | .23^***^ |
| Openness | - | - | - | .08 |
| Conscientiousness | - | - | - | -.14^*^ |
| Emotional stability | - | - | - | -.04 |
| Perceiver characteristics |  |  |  |  |
| Agreeableness | - | - | - | .06 |
| Extraversion | - | - | - | -.003 |
| Openness | - | - | - | -.01 |
| Conscientiousness | - | - | - | .03 |
| Emotional stability | - | - | - | .06 |
| **Random effects** |  |  |  |  |
| Team | .001 | .001 | .001 | .001 |
| Perceiver | .112^***^ | .111^***^ | .115^***^ | .117^***^ |
| Target | .531^***^ | .523^***^ | .467^***^ | .446^***^ |
| Dyad^a^ | .350 | .351 | .350 | .351 |
| Generalized reciprocity | .080^**^ | .076^**^ | .080^**^ | .086^**^ |
| Dyadic reciprocity | -.039 | -.039 | -.039 | -.040 |
|  | **DV: perceived respectful behavior,** β | | | |
|  | Model 0 | Model 1 | Model 2 | Model 3 |
| **Fixed effects** |  |  |  |  |
| Target characteristics |  |  |  |  |
| Agreeableness | - | .04 | - | .02 |
| Extraversion | - | - | .02 | .01 |
| Openness | - | - | - | .01 |
| Conscientiousness | - | - | - | .02 |
| Emotional stability | - | - | - | .01 |
| Perceiver characteristics |  |  |  |  |
| Agreeableness | - | - | - | .11^+^ |
| Extraversion | - | - | - | -.09 |
| Openness | - | - | - | -.01 |
| Conscientiousness | - | - | - | .07 |
| Emotional stability | - | - | - | .03 |
| **Random effects** |  |  |  |  |
| Team | .030 | .029 | .030 | .031 |
| Perceiver | .484^***^ | .483^***^ | .485^***^ | .477^***^ |
| Target | .019 | .018 | .018 | .021 |
| Dyad^a^ | .467 | .467 | .467 | .467 |
| Generalized reciprocity | .003 | -.002 | .004 | -.004 |
| Dyadic reciprocity | -.042 | -.041 | -.042 | -.043 |

*Note.* ^+^*p* < .10, ^*^*p* < .05, ^**^*p* < .01, ^***^*p* < .001; Fixed effects are standardized regression coefficients (obtained by standardizing all predictor and outcome variables; SRM_R app does not provide confidence intervals); random effects can be interpreted as variance attributed to the respective factors; ^a^is confounded with error variance and the software provides no significance test for it.

Figure S1

*Exploratory mediation model, Study 3*

*
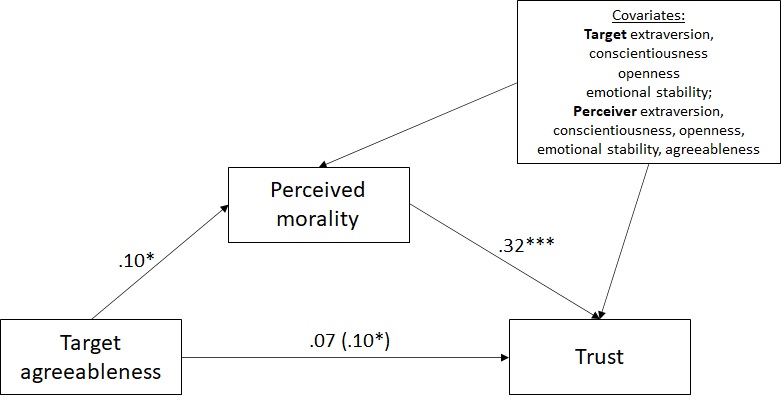
*

Note. ^*^*p* < .05. ^**^*p* < .01. ^***^*p* < .001; The total effect is in the brackets; Path coefficients are standardized regression weights.

**Study 4**

Manipulation text

Experimental condition

“Person 1 is another study participant named Cory. To help Cory make the decision to send you the money or not, we would like to provide Cory with some additional information about you.

Take a moment to think about how Cory will make the decision to send you the money or not. Remember, if Cory sends you the money, it will be tripled. Cory will probably be concerned about whether you will send the money back or keep it.
To help Cory understand what you are like, we would like to ask you to answer several questions about yourself.
For each statement below, think carefully about how you want to be perceived by Cory.

Once you are done, your answers will be shown to Cory. Cory will then make the choice to transfer you the money or not.”

Control condition

“Before we start, we would like to ask you to answer several questions about yourself.
For each statement below, indicate how accurately it describes you.”
